# The association between gut microbiota and postoperative delirium in patients

**DOI:** 10.21203/rs.3.rs-2456664/v1

**Published:** 2023-01-23

**Authors:** Zhongcong Xie, Yiying Zhang, Kathryn Baldyga, Yuanlin Dong, Wenyu Song, Mirella Villanueva, Hao Deng, Ariel Mueller, Timothy Houle, Edward Marcantonio

**Affiliations:** Massachusetts General Hospital; Geriatric Anesthesia Research Unit, Department of Anesthesia, Critical Care and Pain Medicine, Massachusetts General Hospital and Harvard Medical School

**Keywords:** Gut, microbiota, parabacteroides distasonis, prevotell, postoperative delirium

## Abstract

Postoperative delirium is one of the most common postoperative complications in older patients. Its pathogenesis and biomarkers, however, remain largely undetermined. Majority of human microbiota is gut microbiota and gut microbiota has been shown to regulate brain function. Therefore, this study aimed to determine the association between gut microbiota and postoperative delirium in patients. Of 220 patients (65 years old or older) who had a knee replacement, hip replacement, or laminectomy under general or spinal anesthesia, 86 participants were included in the data analysis. The incidence (primary outcome) and severity of postoperative delirium was assessed for two days. Fecal swabs were collected from participants immediately after surgery. The 16S rRNA gene sequencing was used to assess gut microbiota. Using principal component analyses along with a literature review to identify biologically plausible mechanisms, and three bacterials were studied for their associations with postoperative delirium. Of the 86 participants [age 71.0 (69.0–76.0, 25%–75% percentile of quartile), 53% female], ten (12%) developed postoperative delirium. Postoperative gut bacteria *Parabacteroides distasonis* (Odds Ratio [OR] 2.13, 95% Confidence Interval (CI): 1.09–4.17, P = 0.026) was associated with postoperative delirium after adjusting for age and sex. The association between delirium and both *Prevotella* (OR: 0.59, 95% CI: 0.33–1.04, P = 0.067) and *Collinsella* (OR: 0.57, 95% CI: 0.27–1.24, P = 0.158) did not meet statistical significance. These findings suggest that postoperative gut microbiota (e.g., *Parabacteroides distasonis)* may serve as biomarkers in the pathogenesis of postoperative delirium, pending confirmative studies.

## Introduction

Postoperative delirium is one of the most common postoperative complications in older patients ^1^, and is associated with nosocomial complications ^2^, extended hospital stays ^3^, a higher chance of institutional discharge ^4,5^, and increased morbidity ^5–8^ and mortality ^9,10^. It has been reported that the annual healthcare costs in the United States attributable to postoperative delirium are $32.9 billion ^11^. However, there are no targeted interventions for postoperative delirium at present due to a lack of complete understanding of postoperative delirium pathogenesis.

Inflammation, neuroinflammation, and Alzheimer’s disease neuropathogenesis (e.g., tau) have been reported as biomarkers and pathogenesis of postoperative delirium ^11,12^. Specifically, plasma concentrations of c-reactive protein ^13^, neurofilament light ^14^, chitinase-3-like protein 1 ^15^, interleukin 6 ^16^, and metabolites ^17^ have been associated with postoperative delirium in patients.

Previous studies have reported that an elevated preoperative cerebrospinal fluid (CSF) tau/β-amyloid ratio is associated with a higher incidence and greater severity of postoperative delirium in patients ^18^. Changes from preoperative to postoperative day one plasma tau concentrations are associated with the severity of postoperative delirium and are higher in patients who develop postoperative delirium ^19^. In previous studies the preoperative plasma concentrations of tau phosphorylated at threonine 217 (Tau-PT217), and threonine 181 (Tau-PT181) were associated with postoperative delirium in patients undergoing orthopedic surgery ^20^. However, identifying the pathogenesis and biomarkers of postoperative delirium remain largely to be determined, impeding the progress of postoperative delirium research.

Up to 95% of the entire human microbiota is gut microbiota ^21^, and it is well-known that gut microbiota can regulate brain function ^22–25^. Gut microbiota could be associated with changes in immune functions and increase the risk of some human diseases ^26^. Specifically, gut microbiota dysbiosis, an imbalance of gut microbiota associated with an bad outcome, may promote cognitive impairment and Alzheimer’s disease neuropathogenesis ^21,27–30^.

In a previous study of 18 month-old mice, anesthesia and/or surgery was associated with changes in gut microbiota and cognitive impairment 48 hours and 5 to 8 days after the anesthesia/surgery, respectively ^31^. A recent study showed that gut microbiota alteration contributes to developing postoperative cognitive impairment in mice ^32^. Our recent animal study showed that anesthesia/surgery reduced the abundance of gut lactobacillus and induced delirium-like behavior in the 18 months-old mice ^33^. Treatment with lactobacillus or probiotic mitigated the anesthesia/surgery-induced delirium-like behavior in the mice ^33^. These pre-clinical data suggest that gut microbiota may contribute to the pathogenesis and serve as a biomarker of postoperative delirium. However, to our knowledge, no clinical studies have shown the association between gut microbiota and postoperative delirium in human subjects.

Therefore, the objective of the present prospective observational cohort study was to assess the association between the changes in gut microbiota and postoperative delirium in patients. This study aimed testing a hypothesit that changes in gut microbiota were associated with postoperative delirium in patients. The outcomes of the study could be used to further explore why some patients are more vulnerable to developing postoperative delirium and whether we can target gut microbiota to prevent or treat postoperative delirium in patients, ultimately leading to better surgical care for patients.

## Methods

### Study Enrollment

Upon approval by the Mass General Brigham Institutional Review Board, this prospective observational cohort study was performed at Massachusetts General Hospital, Boston, MA, between 2016 and 2020. Patients who were 65 years or older, proficient in English, and scheduled to have an elective knee replacement, hip replacement, or laminectomy under general or spinal anesthesia at the study hospital were included.

Patients were excluded from participation if they had any of the following: (1) past medical history of neurological and psychiatric diseases, including Alzheimer’s disease (AD), other forms of dementia, stroke, or psychosis; (2) severe visual or hearing impairment; (3) current smokers; or (4) taking antibiotics within one week of surgery. Trained clinical research coordinators approached eligible patients for participation during preoperative clinic visits. Written informed consent was obtained at the time of enrollment, prior to the initiation of the study procedures. This manuscript is being reported in accordance with the STrengthening the Reporting of OBservational studies in Epidemiology (STROBE) criteria.

### Anesthesia, Surgery, and Fecal Sample Collection and Measurement

Enrolled participants had a knee replacement, hip replacement, or repair of spinal stenosis under general or spinal anesthesia. All participants received standardized perioperative care, including standard postoperative pain management (e.g., patient-controlled analgesia with hydromorphone). Depth of sedation was at the discretion of the treating provider but was not captured in the current study. There have been no significant changes in the surgery or anesthesia practice since the start of the study. Fecal swabs were collected from participants immediately after surgery. The 16S rRNA gene sequencing was performed by BGI America (Cambridge, MA) as previously described ^33^.

### Determination of Postoperative Delirium

Trained clinical research coordinators interviewed participants to determine the presence or absence of postoperative delirium on postoperative days one and/or two. The Confusion Assessment Measurement (CAM) was used in this study, which is a diagnostic algorithm used to determine the presence or absence of delirium, which has high reliability ^34, 35^. Sixty-four of the 86 participants were assessed on both days, one of the 10 participants with postoperative delirium, and 21 of the 76 participants without postoperative delirium had the CAM on one day. The incidence of postoperative delirium, the primary outcome, was assessed using the CAM once per day between 8:00 am and 12:00 noon. Delirium was defined as present if it occurred on either postoperative day one or day two. The preoperative CAM was not performed in the present study because previous studies ^36, 37^ have shown that participants who underwent elective surgeries had a very low incidence of preoperative delirium.

The secondary outcome was the severity of postoperative delirium, represented by the Memorial Delirium Assessment Scale (MDAS) ^35, 38^, quantifying delirium-related symptoms based on 10 features. Each feature is scored from 0 (best) to 3 (worst symptom) with a maximal score of 30. We determined the MDAS scores for all patients, regardless of whether they had the presence of delirium, based on the results from CAM, on that day. The MDAS score (averaged from both days if two postoperative tests were performed or from one day if only one postoperative test was performed) was used to assess delirium severity independent of the results obtained from CAM. We performed the postoperative MMSE as part of CAM ^34, 39^ and also for MDAS calculation on postoperative day one and/or day two ^39^.

### Dimension-Reduction Algorithm (DASH)

We developed a specialized feature engineering process to reduce comlexity of input variables without losing important information. Specifically, Principal component analysis (PCA) was performed among 740 gut bacteria to reduce these variables’ dimensions (one variable per gut bacteria). The eigenvalue above 3.0 PC component were chosen, which represented 72.8% of the total variance in the dataset. Second, bacteria from the selected PC component were ranked according to contribution index from highest score (e.g., ± 0.12 ) to lowest score (e.g., ± 0.003), resulting in the top 35 gut bacteria. Third, gut bacteria without information at the species level were excluded, resulting in 15 gut bacteria. Fourth, eight gut bacteria were selected from the 15 gut bacteria based on domain expertise that these eight bacteria are associated with human diseases from previous studies. Finally, three gut bacteria (*Parabacteroides distasonis, Prevotella,* and *Collinsella*), but not the other five gut bacteria, were investigated in the present study based on their relevance with inflammation, which contributes to cognitive dysfunction ^40–45^.

### Statistical Analysis

Descriptive statistics were conducted using methods appropriate for the variables under study. Means and standard deviations were used for continuously scaled variables that were normally distributed. Medians and 25th and 75th percentiles were used for skewed or ordinal data. Frequency counts and percentages or proportions were used for categorical variables. Differences in baseline characteristics between those who did and did not develop delirium were assessed with a t-test, Mann-Whitney U-test (for non-normal continuous data), chi-square, or Fisher’s exact test (in the case of small cell counts), as appropriate.

A principal component analysis (PCA) was conducted to reveal hidden patterns across high-dimensional bacteria abundance measurements at the species level. Robust logistic regression was then used to assess the relationship between the three selected gut bacteria and postoperative delirium as the binary outcome. Results are presented as odds ratio (OR) per one unit of relative abundance of gut bacteria change in the biomarker and their associated 95% confidence intervals (CI). Linear regression was used to evaluate the association between bacteria and delirium severity as the contious outcome, with results presented as a mean difference (beta coefficient [β]) and its associated 95% confidence interval. Models were created to adjust for the associations between the biomarkers and outcomes for age and sex for both the primary and secondary outcomes. Variables for adjustment were based on previous studies as deemed clinically relevant.

The present study did not consider multiple comparisons because of the exploratory nature in which the final association model was based on reduced inputs with a small number (e.g., three bacteria) of independent variables according to the statistical principles described before ^46^. All analyses were conducted using R version 4.0.5 statistical software (Vienna, Austria). Where appropriate, all analyses used two-tailed hypothesis testing, with statistical significance interpreted at p < 0.05.

## Results

**Figure 1:**
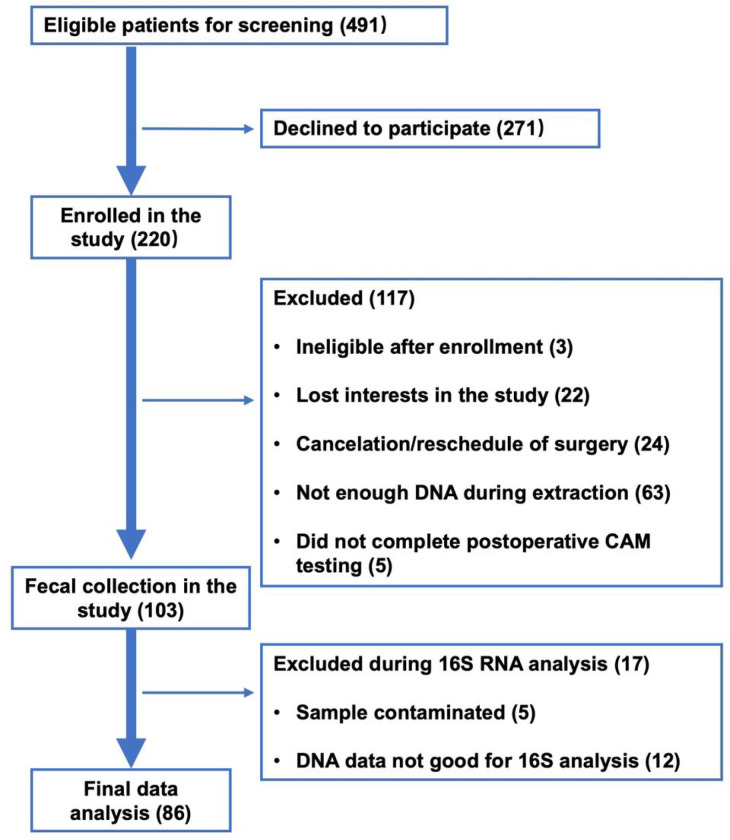
Flow Diagram. The flow diagram shows that 491 participants were screened for the studies, and 220 were initially enrolled. A total of 117 participants were excluded after enrollment, and 103 participants were included in the gut microbiota cohort. During the analysis 17 additional participants were excluded, resulting in 86 participants for the final data analysis.

A total of 491 patients were screened, of which 220 participants were enrolled. A total of 117 participants were excluded, owing to becoming ineligible after enrollment (N = 3), no longer expressing interest in participating (N = 22), cancellation/rescheduling surgery (N = 24), not having enough DNA during extraction (N = 63), or not completing the postoperative CAM testing (N = 5). Thus, 103 participants were included in the gut microbiota cohort. Of these, 17 participants were further excluded due to DNA sample contamination (N = 5) and poor quality of 16S rRNA gene sequencing (N = 12). Thus, 86 participants were included in the data analysis (Fig. 1). There were no significant differences in the demographic characteristics between the participants included (N = 86) and the participants excluded (N = 134; **Supplemental Table 1**). There were no significant complications among participants during the immediate postoperative period.

Ten of the 86 (12%) participants developed postoperative delirium (Table 1). Baseline demographic and clinical characteristics of the 86 participants are presented in Table 1. There were no significant differences in age, gender, ethnicity, surgery type, anesthesia type, or preoperative Mini-Mental State Examination (MMSE) score between the participants with postoperative delirium (N = 10) and those without postoperative delirium (N = 76). The participants who developed postoperative delirium had lower postoperative MMSE scores than the participants who did not develop postoperative delirium (Table 1).

### Postoperative gut microbiota abundances were associated with the incidence of postoperative delirium

**Figure 2:**
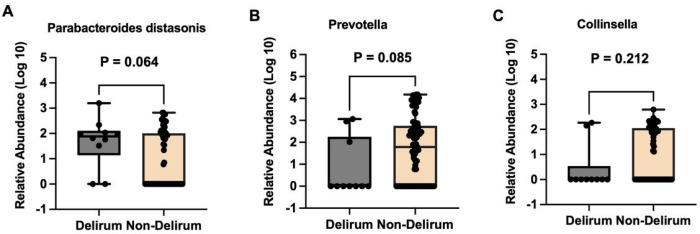
Different postoperative gut bacteria between participants with and without postoperative delirium. Participants who developed postoperative delirium (N = 10) had a higher postoperative relative abundance of gut bacteria of *Parabacteroides distasonis* a lower abundance of postoperative gut bacteria of *Prevotella* (B), but not *Collinsella* (Figure 2C), than the participants who did not develop postoperative delirium (N = 76). The box indicates median (50 th percentile), the first quartile (25 th percentile), and the third quartile (75 th percentile) of the abundances of bacteria.

A total of 740 postoperative gut bacteria were identified and used in the principal component analysis (PCA). The participants with and without postoperative delirium showed a significant difference in the index of principal component 8 (**Supplemental Fig. 1**). Using the methods described above, three gut bacteria were identified, with the association between these bacteria and the incidence and severity of delirium presented in Fig. 2 and Table 2. In unadjusted analyses the abundance of postoperative gut bacteria *Parabacteroides distasonis* was associated with postoperative delirium (OR 1.97, 95% CI: 1.04–3.74, P = 0.038). There was no statistically significant association between the abundances of *Prevotella* (OR 0.62, 95% CI: 0.36–1.07, P = 0.085) or *Collinsella* (OR 0.60, 95% CI: 0.28–1.30, P = 0.197) and postoperative delirium (Table 2).

After the adjustment for age and sex, similar results were observed. The abundance of postoperative *Parabacteroides distasonis* was significantly associated with postoperative delirium (OR 2.13, 95% CI: 1.09–4.17, P = 0.026). Both *Prevotella* (OR: 0.59, 95% CI: 0.33–1.04, P = 0.067) and *Collinsella* (OR 0.57, 95% CI: 0.27–1.24, P = 0.158), were not significantly associated with the incidence of postoperative delirium. Further, the abundance of postoperative *Parabacteroides distasonis, Prevotella* and *Collinsella* were not associated with the severity of postoperative delirium, both before and after adjusting for age and sex (Table 2).

#### Different profile of postoperative gut microbiota in postoperative delirium.

Next, we found that the participants who developed postoperative delirium had a higher postoperative abundance of gut bacteria of Parabacteroides distasonis (1.659 ± 0.984 vs. 0.850 ± 1.080, Mann Whitney U test, P = 0.064, borderline of statistical significance, Fig. 2A), lower abundance of postoperative gut bacteria of Prevotella (0.803 ± 1.321 vs. 1.687 ± 1.459, Mann Whitney U test, P = 0.085, borderline of statistical significance, Fig. 2B), but not Collinsella (0.442 ± 0.932 vs. 0.903 ± 1.040, Mann Whitney U test, P = 0.212, Fig. 2C).

## Discussion

In this prospective observational cohort study, the gut bacteria Parabacteroides distasonis, was associated with the incidence of postoperative delirium in patients. Pending confirmative studies, these data suggest that gut microbiota dysbiosis, the pathogenesis of several brain dysfunction ^22–25^, contributes partially to the development of postoperative delirium. Certain gut bacteria may serve as risk biomarkers, pathogenesis, and potential target of intervention(s) of postoperative delirium in patients.

In this study about 12% of participants developed postoperative. These findings are consistent with many other postoperative delirium clinical investigations ^47–50^, which revealed a 5.1–19.4% incidence of postoperative delirium in patients.

Increases in the abundance of postoperative gut bacteria *Parabacteroides distasonis* were associated with the increases in the incidence of postoperative delirium in the patients after the adjustment of age and sex. Although generally lower in delirious patients, decreases in the abundance of *Prevotella* and *Collinsella* were not significantly associated with delirium. Moreover, patients who developed postoperative delirium had a higher abundance of postoperative gut *Parabacteroides distasonis,* and lower abundance of postoperative gut *Prevotella* and *Collinsella.* These data suggest the contribution of the gut-brain axis to postoperative delirium, and certain gut bacteria can predict postoperative delirium. Future studies will include whether we can target changing the abundance of postoperative gut bacteria, for example, by reducing the abundance of *Parabacteroides distasonis,* to prevent and/or treat postoperative delirium.

*Parabacteroides distasonis* is a bacteria implicated in Crohn’s Disease and ulcerative colitis ^43^ and *Prevotella* is associated with chronic inflammatory disease ^51^. Therefore, future studies should also determine the potential association between postoperative delirium and Crohn’s Disease, ulcerative colitis, and chronic inflammatory disease.

Previous studies have shown that patients who developed postoperative delirium (N = 20) and did not develop postoperative delirium (N = 20) had a different abundance of preoperative gut bacteria ^52^. Specifically, gut bacteria *Proteobacteria, Enterobacteriaceae, Escherichia shigella, Klebsiella, Ruminococcus, Roseburia, Blautia, Holdemanella, Anaerostipes, Burkholderiaceae, Peptococcus, Lactobacillus,* and *Dorea* were abundant in the patients with postoperative delirium, and *Streptococcus equinus* and *Blautia hominis* were abundant in the patients without postoperative delirium ^52^. However, this previous study is different from the current study, as it determined preoperative, not postoperative, gut microbiota; did not establish an association with the incidence of postoperative delirium; and did not assess the severity of delirium with the MDAS. Thus, the previous study did not demonstrate the association between gut microbiota and postoperative delirum in patients. Future studies should include the systematical determination of the association between postoperative delirium and both pre-and postoperative gut microbiota in a larger-scale study.

Interestingly, the present study did not find the associations between the three gut bacteria and the severity of postoperative delirium in patients, as represented by MDAS scores. However, previous studies show that some biomarkers are only associated with the incidence, not severity, of postoperative delirium in patients ^53^.

Notably, the average MDAS score of the participants in the present study was 6.6 (Table 1). Although Breitbart et al. stated that MDAS Scores ≥ 13 indicate the presence of delirium ^38^, the participants in the study by Breitbart et al. included psychiatry consult patients ^38^. Marcantonio et al. showed that the best MDAS cutoff for postoperative delirium was 5 in the participants with surgery for hip fracture repair ^35^. Therefore, it is reasonable that the average MDAS score was 6.6 in the present study.

A strength of the present study included the use of a Dimension-reduction Algorithm in Small Human-datasets (DASH). This method combined a statistical dimensionality reduction algorithm and domain expertise to perform high-throughput data screening and efficiently extract real signals from the noise background. Because of the small sample size and complicated data structure, data-driven methodology alone was insufficient in finding the relationship between gut microbiota and postoperative delirium in patients. Therefore, by infusing our current research knowledge into the data-driven methodology that allowed us to filter through several hundred variables in a small data set. This method can be particularly powerful for analyzing small but high-dimensional patient-level datasets. However, an important component of this approach requires the manual selection of interesting bacteria, which can introduce bias due to subjective opinions. Nevertheless, in the present study, we identified *Parabacteroides distasonis* because it is associated with inflammation-related disorders^43^, and inflammation is associated with postoperative delirium^12^. *Prevotella* was selected because of its association with chronic inflammatory disease ^44^, and *Collinsella* was selected because of its known association with cumulative inflammatory response ^45^.

Limitations of this study included a small sample size at a single center. However, similar biomarker studies (N = 11 ^54^ and N = 14 ^55^) used smaller sample sizes to draw a solid conclusion. In the present study, one hundred and thirty-four of 220 enrolled participants were excluded primarily due to insufficient DNA amounts in the swapped samples and others. However, there were no significant differences in the characteristics between the 86 participants included in the final data analysis and the 134 patients excluded from the study after the enrollment except for anesthesia type (**Supplemental Table 1**). But, previous studies have demonstrated that anesthesia type does not affect postoperative delirium ^47, 56^. In addition, we did not perform preoperative CAM in the participants since these participants had elective cases and the rate of preoperative delirium would be very low based on the findings from previous studies by Mei et al. (0 of 606 participants) ^36^ and Shi et al. (3 of 192 participants) ^37^. Therefore, the presence of postoperative delirium in the study may not be called incident delirium but just postoperative delirium.

In conclusion, in this present proof of concept and system establishment study, patients who developed postoperative delirium had a higher abundance of postoperative gut bacteria *Parabacteroides distasonis* than those who did not develop postoperative delirium. These findings suggest that gut microbiota dysbiosis significantly contributes to postoperative delirium, and may be used to promote more research to prevent or treat postoperative delirium by restoring gut microbiota dysbiosis.

## Figures and Tables

**Table 1:** Demographic characteristics of the participants

	Delirium (N = 10)	No Delirium (N = 76)	P-value
Age, median (25%–75% percentile of quartile)	72.0 (70.5–75.3)	71.0 (69.0–76.8)	0.499
Female, n (%)	4 (40)	42 (46)	0.521
Non-white or Hispanic, n (%)	1 (10)	3 (3.9)	0.289
Education years, median (25%–75% percentile of quartile)	16 (16.0–16.0)	16.4 (16.0–18.0)	0.866
Surgery type, n (%)			
Knee replacement	5 (50)	48 (63)	0.332
Hip replacement	3 (30)	23 (30)	
Spinal stenosis	2 (20)	5 (7)	
Anesthesia type, n (%)			
General	6 (60)	40 (53)	0.661
Spinal	4 (40)	36 (47)	
MMSE, median (25% –75% percentile of quartile)			
Pre-surgery score	29.0 (29.0–30.0)	29.0 (28.0–30.0)	0.734
Post-surgery score	27.5 (26.5–28.6)	29.0 (28.5–30.0)	0.004

MMSE, mini-mental status examination.

**Table 2:** Association between gut bacteria and postoperative delirium*

Presence of Postoperative Delirium **				
	Unadjusted		Adjusted for Age and Sex	
	Odds Ratio (95%CI)	P-Value	Odds Ratio (95% CI)	P-Value
Parabacteroides distasonis	1.97 (1.04 to 3.74)	0.038	2.13 (1.09 to 4.17)	0.026
Prevotella	0.62 (0.36 to 1.07)	0.085	0.59 (0.33 to 1.04)	0.067
Collinsella	0.60 (0.28 to 1.30)	0.197	0.57 (0.27 to 1.24)	0.158
The Severity of Postoperative Delirium ***				
	Unadjusted		Adjusted for Age and Sex	
	β Coefficient (95% CI)	P-Value	β Coefficient (95% CI)	P-Value
Parabacteroides distasonis	0.23 (−0.06 to 0.52)	0.123	0.21 (−0.13 to 0.55)	0.226
Prevotella	−0.12 (−0.32 to 0.08)	0.242	−0.11 (−0.32 to 0.09)	0.288
Collinsella	−0.14 (−0.45 to 0.17)	0.383	−0.10 (−0.45 to 0.24)	0.564

* Models were created to adjust the associations between the bacteria and outcomes for age and sex based on previous studies as deemed clinically relevant.

** Results are presented as odds ratio (OR) per one unit change in gut bacteria value and its associated 95% confidence intervals (CI).

*** Results are presented as the beta coefficient (β) per one unit change in gut bacteria value and its associated 95% CI.
